# Tuberculosis amongst foreign-born and nationals: different delays, different risk factors

**DOI:** 10.1186/s12879-021-06635-1

**Published:** 2021-09-08

**Authors:** Marta Moniz, Patrícia Soares, Andreia Leite, Carla Nunes

**Affiliations:** 1 NOVA National School of Public Health, Public Health Research Centre, Universidade NOVA de Lisboa, Lisbon, Portugal; 2 Comprehensive Health Research Centre (CHRC), Universidade NOVA de Lisboa, Lisbon, Portugal

**Keywords:** Foreign-born, Tuberculosis, Diagnosis delay, Risk factors

## Abstract

**Background:**

Delay in Tuberculosis (TB) diagnosis affects foreign-born and nationals in different ways, especially in low-incidence countries. This study characterises total delay and its components amongst foreign-born individuals in Portugal. Additionally, we identify risk factors for each type of delay and compare their effects between foreign-born and nationals.

**Methods:**

We analysed data from the Portuguese TB surveillance system and included individuals with pulmonary TB (PTB), notified between 2008 and 2017. We described patient, healthcare, and total delays. Cox regression was used to identify factors associated with each type of delay. All analyses were stratified according to the origin country: nationals (those born in Portugal) and foreign-born.

**Results:**

Compared with nationals, foreign-born persons presented statistically significant and longer median total and patient delays (Total: 67 vs. 63; Patient: 44 vs. 36 days), and lower healthcare services delays (7 vs. 9 days). Risk factors for delayed diagnosis differed between foreign-born and nationals. Being unemployed, having drug addiction, and having comorbidities were identified as risk factors for delayed diagnosis in national individuals but not in foreigners. Alcohol addiction was the only factor identified for healthcare delay for both populations: foreign-born (Hazard Ratio 1.34 [95% confidence interval 1.17;1.53]); nationals (Hazard Ratio 1.20 [95% confidence interval 1.13;1.27]).

**Conclusions:**

Foreign-born individuals with PTB take longer to seek health care. While no specific risk factors were identified, more in-depth studies are required to identify barriers and support public health intervention to address PTB diagnosis delay in foreign-born individuals.

**Supplementary Information:**

The online version contains supplementary material available at 10.1186/s12879-021-06635-1.

## Background

Tuberculosis (TB) is a curable disease but persists amongst the top 10 causes of death worldwide and was the number one infectious killer worldwide in 2018. TB thus remains a major public health issue in the twenty-first century [1]. TB infection can affect different organs, but it typically affects the lungs (pulmonary TB), becoming contagious and spreading via person-to-person transmission [2].

Appropriate and timely diagnosis is a cornerstone to TB control; delays in detecting TB cases are related to increased transmission, severity, and mortality rates [3–6]. These can be defined as the time between symptoms onset and the diagnosis/beginning of treatment (total delay) and be divided into patient delay (time between symptoms onset and the first contact with health services) and healthcare services delay (time between the first contact with health services and diagnosis/beginning of treatment) [3, 4]. Ideally, the total delay should take no longer than 3–4 weeks [4, 7]. However, in Portugal the median delay is 80 days [8].

TB affects some areas and population groups disproportionally, with immigrants in urban areas presenting a higher incidence than nationals [5, 6, 9–13]. Moreover, the overall decrease of TB cases in Europe in recent years [2, 8] has not been reflected in immigrants [2, 12–14]. Portugal follows a similar trend, with notifications of TB cases amongst foreign-born individuals increasing, reaching 20.2% from overall TB cases in 2018 [8]. (15.9% in 2014 [15]; 18.4% in 2016 [16]; 19.2% in 2017 [8]) [9, 12, 17–20]. In recent years the immigrant population in Portugal has also been growing annually (6.0% in 2017 [21]; 13.9% in 2018 [20]), coming mostly from other European and Portuguese speaking countries [20]. Additionally, foreign-born individuals mainly enter Portugal with the purpose of settling, with refugees and asylum seekers accounting for very few [20]. Screening upon entrance is not undertaken [22].

The immigrant status is a well-known risk factor not only for TB disease but also for the diagnosis delay, with immigrants presenting significantly longer patient delays, which could be considered a proxy for access to healthcare services [3, 5, 7, 11–13, 18, 23–25]. Studies show that immigrants seek medical care at an advanced or intermediate TB stage [23, 24, 26–28]. Delayed seeking of medical care amongst immigrants is often attributed to an interplay of several factors, such as living and working conditions, legal status, poor access to services, discrimination [24], poor knowledge regarding TB, availability of appropriate awareness materials, communication campaigns in different languages, literacy levels, and different sociocultural beliefs (i.e., preferring traditional healers) [24, 29]. However, there are limited studies addressing the risk factors for diagnosis delays in this specific population and to the best of our knowledge, none has been conducted in Portugal. With a rising number of cases amongst foreign-born individuals and persistent delays in the diagnosis, an analysis of the factors associated with delays is required to design and support public health intervention.

## Methods

This study characterises total delay and its components amongst foreign-born individuals in Portugal. We also identify risk factors for each delay type and compare the effects of those factors between foreign-born and nationals. In this study we used the individuals born outside Portugal (foreign-born) as a proxy for immigrant status.

We used data from the national surveillance system for TB (SVIG-TB [30]) between 2008 and 2017, and analysed all PTB cases notified in Portugal identified through passive case finding (i.e., presenting symptoms). Only PTB cases were included since it presents the most common and the only contagious form of the disease. Finally, only individuals with symptoms were included in the analysis in order to ascertain the time between the onset of symptoms and diagnosis [3, 4].

The outcomes under analysis were patient delay, healthcare services delay, and total delay, all measured in days. Diagnosis delay (total delay) was defined as the time between the date of symptoms onset and the diagnosis date. Patient delay was defined as the time between the date of symptoms onset and the first appointment, and healthcare services delay was defined as the time between the first medical appointment and the diagnosis date. The variables included in the analyses were selected according to those identified in the literature [5, 14, 31–33], and included sex (male/female); age at diagnosis (in years); unemployment (yes/no); prison inmate (yes/no); homelessness (yes/no); community residence (yes/no); alcohol addiction (subjective information based on CAGE score [34]) (yes/no); drug addiction (excluding occasional use and requiring withdrawal symptoms) (yes/no); HIV positivity (yes/no); comorbidities (non-respiratory/respiratory) (yes/no). Non-respiratory comorbidities included kidney failure on dialysis, cancer, diabetes, liver disease, and respiratory comorbidities included chronic obstructive pulmonary disease, silicosis, interstitial pulmonary/lung disease.

As we intended to compare delays amongst foreign-born and nationals, all analyses were stratified based on the country of origin: those born outside of Portugal were considered as foreign-born, while individuals born in Portugal were deemed nationals.

We performed a descriptive analysis to characterise PTB cases in foreign-born individuals and nationals. The median test for independent samples was used to compare the medians of delays in both groups. A chi-square independence test was performed to identify the association between the origin country and the independent variables. A Cox regression model was used to identify factors associated with the delays in foreign-born individuals and nationals. The initial model included sex and age, and the remaining variables were selected using backward selection. Only complete cases were included. Significance level was set at 5%. The model goodness-of-fit was assessed using concordance [35]. All statistical analyses were performed using SPSS (version 26). As data used in these analyses are from an official national surveillance system and were previously anonymized, ethics committee approval and informed consent were not required.

## Results

From 2008 to 2017, 15,359 cases of PTB identified through passive finding were notified to SVIG-TB. Data were validated, and 460 cases were removed due to inconsistencies. The final sample size included 14,899 cases, 15.5% of which were born outside Portugal (Table 1). Foreign-born PTB cases were mainly male (66.7%) and aged 25–54 years old (70.7%). Foreign-born individuals were more frequently HIV-positive (9.4% vs. 18.7%), homeless people (1.5% vs. 2.9%) and people living in community residencies (3.1% vs. 4.1%). Foreign-born individuals had lower frequency of comorbidities (non-respiratory—16.0% vs. 12.5%; respiratory—6.7% vs. 1.6%), lower frequency of alcohol (15.2% vs. 13.6%) and drug addiction (11.1% vs. 7.4%), and lower frequency of imprisonment (1.9% vs. 1.7%). Statistically significant differences (p < 0.005) were identified for all variables except unemployment, alcohol addiction, and incarceration.

**Table 1:** Sociodemographic and clinical characteristics of nationals and foreign-born with pulmonary tuberculosis notified in SVIG-TB (2008–2017)

Variables	Nationals (N,%)	Foreign-born (N,%)	p-value^a^
	N = 12,596 (84.5%)	N = 2303 (15.5%)	
*Sex*			< 0.001
Male	8934 (70.9%)	1537 (66.7%)	
Female	3662 (29.1%)	766 (33.3%)	
*Age at diagnosis*			< 0.001
0–4	59 (0.5%)	9 (0.4%)	
5–14	72 (0.6%)	26 (1.1%)	
15–24	1057 (8.4%)	308 (13.4%)	
25–34	1844 (14.7%)	518 (22.6%)	
35–44	2683 (21.3%)	646 (28.1%)	
45–54	2572 (20.4%)	459 (20.0%)	
55–64	1724 (13.7%)	212 (9.2%)	
≥ 65	2573 (20.4%)	119 (5.2%)	
*Unemployed* > *24 months*			0.63
No	10,609 (84.2%)	1949 (84.6%)	
Yes	1987 (15.8%)	354 (15.4%)	
*Alcohol addiction*			0.05
No	10,031 (79.6%)	1868 (81.1%)	
Yes	1910 (15.2%)	313 (13.6%)	
Unknown	655 (5.20%)	122 (5.3%)	
*Drug addiction*			< 0.001
No	10,668 (84.7%)	2016 (87.5%)	
Yes	1393 (11.1%)	170 (7.4%)	
Unknown	535 (4.3%)	117 (5.1%)	
*Imprisoned*			0.61
No	12,005 (95.3%)	2186 (94.9%)	
Yes	240 (1.9%)	40 (1.7%)	
Unknown	351 (2.8%)	77 (3.3%)	
*Homelessness*			< 0.001
No	12,041 (95.6%)	2159 (93.7%)	
Yes	189 (1.5%)	67 (2.9%)	
Unknown	366 (2.9%)	77 (3.3%)	
*Community residence*			0.01
No	11,806 (93.7%)	2113 (91.7%)	
Yes	396 (3.1%)	95 (4.1%)	
Unknown	394 (3.1%)	95 (4.1%)	
*Comorbidities*			< 0.001
No	9606 (77.3%)	1936 (85.9%)	
Non-respiratory^b^	1984 (16.0%)	281 (12.5%)	
Respiratory^c^	835 (6.7%)	36 (1.6%)	
*HIV*^d^*serology*			< 0.001
No	10,180 (80.8%)	1668 (72.4%)	
Yes	1179 (9.4%)	431 (18.7%)	
Unknown	1237 (9.8%)	204 (8.9%)	

^a^Comparison between origin country and the independent variable through the chi-square independence test

^b^Includes kidney failure on dialysis, cancer, diabetes, liver disease

^c^Includes chronic obstructive pulmonary disease, silicosis, interstitial pulmonary/lung disease

^d^Human immunodeficiency virus

Median patient and total delay were higher in foreign-born individuals (44 vs. 36 days; 68 vs. 64 days, respectively, both p < 0.001), while the median healthcare services delay was lower for this population (7 vs. 9 days, p = 0.005), compared with nationals, all of them statistically different.

Table 2 shows the results for the multivariable Cox regression model, adjusted for sex and age and the variables identified through backward selection. The outcome corresponds to PTB diagnosis, hence an HR above 1 indicates shorter delays as it represents a higher risk of having the event. Among nationals, being addicted to alcohol was associated with longer patient delay and having HIV co-infection was associated with shorter patient delay. For the same population, living in a community residence and being addicted to alcohol were associated with shorter healthcare services delay, but having comorbidities was associated with longer healthcare services delay. Being homeless and HIV co-infected were associated with shorter total delay, while being addicted to drugs was associated with longer total delay. Among foreign-born and for patient and total delays, no factors apart from sex or age were identified as statistically significant. Alcohol addiction was the only factor identified to be associated with shorter healthcare services delay.

**Table 2:** Hazard ratios for patient, healthcare services, and total delays and individual variables, adjusted for sex and age-group (multivariable analysis)

Variables	Patient delay—HR (CI95%)^g^				Healthcare services delay—HR (CI95)^g^				Total delay—HR (CI95%)^g^			
	NT^a^ (0.5^f^)	p-value	FB^b^ (0.50^f^)	p-value	NT^a^ (0.57^f^)	p-value	FB^b^ (0.56^f^)	p-value	NT^a^ (0.55^f^)	p-value	FB^b^ (0.53^f^)	p-value
Unemployment > 24 months^c^	0.93 (0.87;0.98)	0.01	–	–	1.08 (1.02;1.14)	0.01	–	–	–	–	–	–
Imprisoned^c^	–	–	–	–	–	–	–	–	–	–	–	–
Homelessness^c^	–	–	–	–	–	–	–	–	1.30 (1.09;1.54)	< 0.001	–	–
Community residence^c^	–	–	–	–	1.23 (1.10;1.39)	< 0.001	–	–	–	–	–	–
Alcohol addiction^d^	0.90 (0.84;0.95)	< 0.001	–	–	1.20 (1.13;1.27)	< 0.001	1.34 (1.17;1.53)	< 0.001	–	–	–	–
Drug addiction^c^	–	–	–	–	–	–	–	–	0.93 (0.87;1.00)	0.05	–	–
HIV^c,h^	1.24 (1.15;1.33)	< 0.001	–	–	–	–	–	–	1.30 (1.21;1.41)	< 0.001	–	–
Comorbidities												
Non-respiratory^c,d^	–	–	–	–	0.92 (0.87;0.97)	< 0.001	–	–	–	–	–	–
Respiratory^c,e^	–	–	–	–	0.90 (0.83;0.98)	0.01	–	–	–	–	–	–

^a^Nationals (born in Portugal)

^b^Foreign-born (born outside Portugal)

^c^Reference class “no”

^d^Includes kidney failure on dialysis, cancer, diabetes, liver disease

^e^Includes chronic obstructive pulmonary disease, silicosis, interstitial pulmonary/lung disease

^f^Concordance

^g^Hazard ratio with 95% confidence interval

^h^Human immunodeficiency virus

## Discussion

This study characterises the PTB delay amongst foreign-born persons and nationals, considering sociodemographic and clinical information, and compares the results between these two groups. Our analysis shows that both the median of total delay and patient delay are higher for foreign-born individuals than amongst nationals (67 vs. 63 days and 44 vs. 36 days, respectively), while the median healthcare services delay is lower (7 vs. 9 days). These results are in line with other European countries and international literature [3–5, 18, 36]. While there is no period of time between symptoms onset and diagnosis has been established that is deemed to be acceptable, from a PTB control point of view, such a time-lapse should be no more than 4 weeks (about 28 days) [4, 7, 36]. Our study reveals a delay of more than twice that duration, especially among foreign-born. We view this finding as a call for action.

Considering patient delay as a proxy for healthcare services access and the greater patient delay for foreign-born, we speculate that foreign-born persons might face barriers while trying to access healthcare services, possibly contributing thereby to advanced PTB stages upon consultation [3, 18, 23, 31, 37]. A systematic review of qualitative studies concerning TB in immigrants identified numerous reasons contributing to the healthcare services delay, including misconceptions about TB, lack of familiarity with the local language, lack of knowledge of free healthcare, inability to take off from work to attend a clinic appointment, and cultural differences with “Western” medical services [29]. This may suggest that these types of individual issues, such as knowledge of their health rights, cultural beliefs, and/or TB knowledge should be addressed to help identify their influence in the seeking of healthcare services by foreign-born persons diagnosed with TB. In addition, foreign-born persons may have certain beliefs and concepts about disease and health based on their ethnic and cultural background, influencing how they understand signs and symptoms [10, 23]. The role of community health workers could be of extreme importance to bridge the gap between foreign communities and healthcare services, addressing some barriers previously mentioned such as clarifying health rights and dispelling misconceptions regarding TB disease. Additionally, as the risk factors for nationals do not appear to be the same as those for foreign-born individuals, tailored studies including a qualitative component are needed to understand what is causing the delays amongst foreign-born individuals. Healthcare services delay (between first appointment and diagnosis/beginning of treatment), however, is lower in foreign-born individuals, which might be explained through the greater suspicion for TB infection in this population, which is known by health professionals [5, 38]. These results corroborate previous studies from high-income countries, where the patient delay is usually longer than healthcare services delay [4, 39].

As also reported in other studies, foreign-born individuals were younger, had a higher percentage of HIV co-infection, and less alcohol and drug addiction [10, 12, 37]. The age difference between foreign-born and nationals diagnosed with pulmonary TB may be due to the difference in age profile between foreign-born and nationals or might be related to the natural history of TB in the origin country [12, 37]. Studies related to TB amongst immigrants in Europe have showed that this population is disproportionally affected by HIV-TB co-infection and that the risk of substance abuse is higher in nationals [10, 12, 37]. This can contribute to longer patient delays for foreign-born individuals. These individuals also had fewer comorbidities, which is consistent with the literature reporting that foreign-born persons have a lower risk of having chronic pathologies when compared to nationals [37].

The risk factors for nationals showed results that are consistent with findings reported in the literature [3, 5]. Homeless and individuals with an HIV diagnosis had shorter total delays than individuals not homeless and without an HIV diagnosis, for nationals. These results are in line with the literature that suggests that these characteristics are generally associated with shorter total delay, possibly because they are well-known risk factors for TB disease [3, 5]. The main differences between foreign-born and nationals are between patient and healthcare services delay [3, 31]. The risk factors identified as associated with delays in nationals, were not individually associated with delays in foreign-born individuals.

We also found that foreign-born individuals with comorbidities had higher healthcare services delay, although this association was only significant in the crude analysis (Additional file 1: Table S1), which is consistent with the literature [3]. National individuals with comorbidities had longer healthcare services delay, which might be due to similar symptoms presented by PTB patients and patients with other respiratory diseases, which may mislead health professionals [3]. In our study, having HIV infection seems to have divergent effects in these two populations, increasing the healthcare services delay in foreign-born and decreasing it for nationals [31]. However this results were only significant for nationals in the crude analysis (Additional file 1: Table S1), thus further studies are necessary. This is possibly due to linguistic or cultural barriers that may delay the diagnosis, even after the first contact [31].

The absence of statistical significance for some variables in the regression analysis, especially concerning the delays in foreign-born, might be due to lack of power as a consequence of sample size in this population, since incarceration, homelessness, living in a community residence, and having respiratory comorbidities have lower frequencies in foreign-born individuals (n < 100). Also, we can speculate that this is due to the existence of other factors associated with PTB diagnosis for foreign-born that are different from the commonly known factors described in the literature that also contribute to the delays and that may not be collected at the time of notification. Overall, this suggests the need for in-depth studies in the foreign-born population with PTB in order to identify specific risk factors that may contribute to the longer time until seeking healthcare services by this population.

A potential limitation of this study is recall bias, as the patient delay depends on a self-reported variable (symptoms onset). This is likely to have underestimated the patient delay, as patients might tend to place the beginning of symptoms closer to the diagnosis. Even so, this is unlikely to have affected the associations identified as it is constant across notified cases. Collection of diagnosis and beginning of treatment dates can also pose limitations, since we did not have access to information that allowed us to differentiate between the two dates. Exclusion due to inconsistencies represented a very small number of individuals and is therefore unlikely to affect our results. In addition, individuals were deemed as foreign-born based on the country of origin and we did not consider how long they had lived in Portugal, which could impact the results, as some studies suggest [28, 36]. However, we performed an exploratory analysis with the time of stay in Portugal that showed foreign-born PTB cases had lived in Portugal for a median of 10 years. Additionally, it is not known if they had PTB prior to entering the country.

This study also has several strengths. We used data from SVIG-TB, a well-established nationwide surveillance system. To the best of our knowledge, this is the first study in Portugal that analysed diagnosis delay based on the origin country. Furthermore, we analysed patient and healthcare services delays separately, as factors affecting each of these dimensions could be different. Overall, these aspects afford us a better understanding of PTB delays amongst foreign-born, by showing that this population takes longer to have the final contact with healthcare services, but that from this point on, the beginning of treatment comes more quickly. These findings enable oriented actions to tackle this difference, i.e., studying the factors that are preventing foreign-born from getting timely care. Additionally, our results suggest that the factors contributing to the diagnostic delay in this population might not currently be obtained by the current notification system. There might, therefore, be alternative risk factors contributing to the delay in this population that need to be addressed.

## Conclusions

This study identified different risk factors for patient and healthcare services delay amongst foreign-born persons and nationals, highlighting the importance to analyse each component of PTB diagnosis delay. Foreign-born have greater patient delay compared to nationals with different risk factors, and thus tailored interventions should be implemented to guarantee timely access to healthcare services in this population. Additionally, the fact that few of the common risk factors associated with PTB were associated with delay in foreign-born could imply the existence of specific factors contributing to the delay in this population that need to be identified and addressed. Therefore, specific studies should be conducted to provide insightful information related to the specific risk factors associated with diagnosis delay of PTB in foreign-born persons.

## Supplementary Information

**Additional file 1: Table S1.** Hazard ratios for patient, healthcare services and total delays and individual variables (multivariable analysis)—crude hazard ratios.

## Data Availability

The data that support this study are the property of *Programa Nacional de Controle da Tuberculose* (National Tuberculosis Control Program), property of *Direção-Geral da Saúde* (Directorate General of Health—DGS) and restrictions apply to the availability of these data, which were used under license for the current study, and so are not publicly available. Data are, however, available from the corresponding author upon reasonable request and with permission of the DGS.

## Abbreviations

TB: Tuberculosis
PTB: Pulmonary tuberculosis
SVIG-TB: *Sistema de Vigilância da Tuberculsose* (Surveillance System for Tuberculosis)
