# Supplementary material for: Tuberculosis amongst foreign-born and nationals: different delays, different risk factors
Source: BMC Infect Dis. 2021 Sep 8;21:934. doi: 10.1186/s12879-021-06635-1 (PMC8427946; doi:10.1186/s12879-021-06635-1)
Supplement: Supplementary file 1 — Additional file 1: Table S1. Hazard ratios for patient, healthcare services and total delays and individual variables (multivariable analysis)—crude hazard ratios. [file 12879_2021_6635_MOESM1_ESM.docx]

Additional material

Microscopy and culture dates allowed to identify inconsistencies in the date of symptom onset and diagnosis date, underestimating the patient delay and in the healthcare services. therefore, for patient delay were excluded cases in which symptoms onset dates were inconsistent with microscopy and culture dates, for healthcare services delay were excluded cases in which dates of first appointment that were inconsistent with microscopy and culture dates and for global delays were excluded cases in which symptoms onset dates were inconsistent with microscopy and culture dates.

Table S1 – Hazard ratios for patient, healthcare services and total delays and individual variables (multivariable analysis) – crude hazard ratios.

| *Variables* | *Patient delay – HR (CI95%)^g^* | | | | *Healthcare services delay*  *– HR (CI95%)^g^* | | | | *Total delay – HR (CI95%)^g^* | | | |
| --- | --- | --- | --- | --- | --- | --- | --- | --- | --- | --- | --- | --- |
|  | *NT^a^* | *p-value* | *FB^b^* | *p-value* | *NT^a^* | *p-value* | *FB^b^* | *p-value* | *NT^a^* | *p-value* | *FB^b^* | *p-value* |
| *Male^c^* | 0.98 (0.94;1.02) | 0.306 | 0.98  (0.89;1.08) | 0.683 | 1.15 (1.11;1.20) | <0.001 | 1.24 (1.13;1.37) | <0.001 | 1.10 (1.05;1.14) | <0.001 | 1.10 (1.00;1.20) | 0.047 |
| *Age group* |  | | | | | | | | | | | |
| *0-14* | 1.18 (0.96;1.44) | 0.117 | 0.91 (0.58;1.42) | 0.681 | 1.46 (1.20;1.76) | <0.001 | 0.92 (0.62;1.38) | 0.688 | 1.61 (1.33;1.95) | <0.001 | 0.98  (0.64;1.49) | 0.906 |
| *15-24* | 0.98 (0.91;1.06) | 0.658 | 0.95 (0.74;1.22) | 0.683 | 1.55 (1.43;1.67) | <0.001 | 1.42 (1.13;1.79) | 0.003 | 1.39 (1.29;1.50) | <0.001 | 1.35 (1.07;1.70) | 0.011 |
| *25-34* | 0.88 (0.82;0.94) | <0.001 | 0.89 (0.71;1.13) | 0.347 | 1.46 (1.37;1.55) | <0.001 | 1.25 (1.01;1.56) | 0.044 | 1.20 (1.13;1.28) | <0.001 | 1.19 (0.95;1.48) | 0.125 |
| *35-44* | 0.89 (0.84;0.95) | <0.001 | 0.85 (0.67;1.07) | 0.155 | 1.46 (1.38;1.55) | <0.001 | 1.29 (1.04;1.60) | 0.020 | 1.21 (1.14;1.29) | <0.001 | 1.10 (0.89;1.37) | 0.370 |
| *45-54* | 0.82  (0.77;0.88) | <0.001 | 0.89 (0.70;1.13) | 0.330 | 1.38 (1.30;1.46) | <0.001 | 1.27 (1.02;1.59) | 0.033 | 1.10  (1.04;1.17) | 0.002 | 1.11 (0.89;1.38) | 0.364 |
| *55-64* | 0.83  (0.78;0.89) | <0.001 | 0.79 (0.61;1.02) | 0.074 | 1.25 (1.16;1.33) | <0.001 | 1.20 (0.94;1.53) | 0.147 | 1.00 (0.94;1.07) | 0.986 | 0.98 (0.77;1.26) | 0.869 |
| *≥65* | Ref. | Ref. | Ref. | Ref. | Ref. | Ref. | Ref. | Ref. | Ref. | Ref. | Ref. | Ref. |
| *Unemployment > 24 months^d^* | 0.90 (0.86;0.95) | <0.001 | 0.93 (0.82;1.06) | 0.267 | 1.21 (1.15;1.28) | <0.001 | 1.12 (0.99;1.26) | 0.068 | 1.02  (0.97;1.08) | 0.379 | 0.97 (0.86;1.10) | 0.656 |
| *Imprisoned^d^* | 1.06 (0.87;1.28) | 0.578 | 1.25 (0.79;1.96) | 0.343 | 0.95 (0.80;1.13) | 0.564 | 0.84 (0.57;1.24) | 0.381 | 1.12 (0.96;1.31) | 0.145 | 1.09 (0.75;1.60) | 0.645 |
| *Homelessness^d^* | 1.03 (0.88;1.22) | 0.695 | 1.05 (0.80;1.39) | 0.710 | 1.44 (1.24;1.67) | <0.001 | 1.09 (0.85;1.41) | 0.498 | 1.31 (1.12;1.54) | 0.001 | 1.16 (0.88;1.54) | 0.290 |
| *Community residence^d^* | 1.12 (0.99;1.25) | 0.063 | 1.05 (0.83;1.33) | 0.692 | 1.16 (1.04; 1.30) | 0.007 | 1.20 (0.96;1.50) | 0.104 | 1.16 (1.04;1.30) | 0.008 | 1.21 (0.96;1.52) | 0.108 |
| *Alcohol addiction^d^* | 0.85 (0.80;0.89) | <0.001 | 0.91 (0.80;1.04) | 0.151 | 1.28 (1.21;1.34) | <0.001 | 1.36 (1.20;1.55) | <0.001 | 1.01 (0.96;1.06) | 0.705 | 1.08 (0.95;1.22) | 0.257 |
| *Drug addiction^d^* | 0.97 (0.91;1.03) | 0.315 | 0.97 (0.82;1.16) | 0.770 | 1.23 (1.16;1.30) | <0.001 | 1.21 (1.02;1.44) | 0.025 | 1.12 (1.05;1.19) | <0.001 | 1.14 (0.97;1.35) | 0.120 |
| *HIV^d,h^* | 1.19 (1.11;1.27) | <0.001 | 1.02 (0.91;1.15) | 0.726 | 1.17 (1.10;1.25) | <0.001 | 0.99 (0.89;1.12) | 0.919 | 1.32 (1.24;1.41) | <0.001 | 1.07 (0.96;1.20) | 0.228 |
| *Comorbidities* |  |  |  | | | | | | |  |  |  |
| *Non-respiratory^e^* | 1.07 (1.01;1.13) | 0.023 | 0.96 (0.84;1.11) | 0.614 | 0.87 (0.82;0.91) | <0.001 | 0.92 (0.81;1.06) | 0.241 | 0.96 (0.91;1.01) | 0.096 | 0.90 (0.79;1.04) | 0.145 |
| *Respiratory^f^* | 1.04 (0.96;1.13) | 0.323 | 1.06 (0.73;1.53) | 0.777 | 0.83 (0.76;0.89) | <0.001 | 0.92 (0.64;1.32) | 0.653 | 0.89 (0.82;0.96) | 0.003 | 0.89 (0.62;1.26) | 0.502 |

**Notes:** ^a^Nationals (born in Portugal); ^b^Foreign-born (born outside Portugal)**;** ^c^Reference class is being “female”; ^d^reference class “no”; ^e^includes kidney failure on dialysis, cancer, diabetes, liver disease; ^f^includes chronic obstructive pulmonary disease, silicosis, interstitial pulmonary/lung disease; ^g)^Hazard ratio with 95% confidence interval; ^h^Human Immunodeficiency Virus.
